# Electrochemical Reduction of CO_2_ to Formate on Easily Prepared Carbon-Supported Bi Nanoparticles

**DOI:** 10.3390/molecules24112032

**Published:** 2019-05-28

**Authors:** Beatriz Ávila-Bolívar, Leticia García-Cruz, Vicente Montiel, José Solla-Gullón

**Affiliations:** Instituto de Electroquímica, Universidad de Alicante, Apartado 99, 03080 Alicante, Spain; beatriz.bolivar@ua.es (B.Á.-B.); leticia.garcia@ua.es (L.G.-C.); vicente.montiel@ua.es (V.M.)

**Keywords:** electrocatalysis, CO_2_ reduction, Bi electrodes, formate

## Abstract

Herein, the electrochemical reduction of CO_2_ to formate on carbon-supported bismuth nanoparticles is reported. Carbon-supported Bi nanoparticles (about 10 nm in size) were synthesized using a simple, fast and scalable approach performed under room conditions. The so-prepared Bi electrocatalyst was characterized by different physicochemical techniques, including transmission electron microscopy, X-ray photoelectron spectroscopy, and X-ray diffraction and subsequently air-brushed on a carbon paper to prepare electrodes. These electrodes were characterized by scanning electron microscopy, energy-dispersive X-ray spectroscopy and also by cyclic voltammetry. Finally, CO_2_ electroreduction electrolyses were performed at different electrode potentials for 3 h. At the optimal electrode potential (−1.6 V vs AgCl/Ag), the concentration of formate was about 77 mM with a faradaic efficiency of 93 ± 2.5%. A 100% faradaic efficiency was found at a lower potential (−1.5 V vs AgCl/Ag) with a formate concentration of about 55 mM. In terms of stability, we observed that after about 70 h (in 3 h electrolysis experiments at different potentials), the electrode deactivates due to the gradual loss of metal as shown by SEM/EDX analyses of the deactivated electrodes.

## 1. Introduction

The concentration of CO_2_ in the atmosphere is reaching extremely high values (>410 ppm) as consequence of the industrial activity and the combustion of fossil fuels [1]. In this regard, numerous research groups have focused their efforts on the development of different methods including chemical, photochemical, electrochemical, and biological approaches, among others, not only to significantly reduce such high CO_2_ concentration (thus, mitigating the greenhouse effect and climate change) but also to valorise it into more valuable chemicals [2].

Among the different alternatives, the electrochemical approach to efficiently transform CO_2_ into value-added chemicals and fuels is considered one of the most promising ways of CO_2_ valorisation [3,4]. As described in previous contributions, it is possible to generate different products selectively as a function of different experimental conditions including the nature of the electrocatalytic materials, among many other experimental parameters [5,6,7,8,9]. Additionally, the possible coupling with renewable energy sources such as wind and/or solar energy, it allows the electrochemical CO_2_ reduction (ECO_2_R) process to be considered as an eco-friendly approach. However, due to its particular structure, CO_2_ is quite thermodynamically and kinetically stable, and it requires usually high cathodic overpotentials to produce C1, C2 and even C3 compounds. Due to these high overpotentials, the hydrogen evolution reaction (HER) is its serious competitive reaction in aqueous solution. Additionally, the ECO_2_R displays a complex reaction mechanism with multiple electron transfer processes with manifold coupled consecutives intermediates [10]. Also, as previously stated, the product selectivity and efficiency of the ECO_2_R are strongly affected by a number of parameters including the chemical nature of the electrocatalysts [11,12,13,14], the particle shape/ surface structure [7,8,15,16,17,18,19,20,21,22,23,24,25], atomic composition [24,26,27,28,29,30,31], and particle size [32,33,34,35], among others. Consequently, several electrocatalysts [5,7,8,36,37] as well as experimental conditions such as temperature and pressure [5], pH [38,39], and electrolyte composition [5,40,41,42] have been explored.

In aqueous electrolyte and depending on the electroactive material, CO_2_ can be reduced to a wide number of products including carbon monoxide (CO), methane (CH_4_), ethylene (C_2_H_4_), methanol (CH_3_OH), ethanol (CH_3_CH_2_OH), and other high added value products such as formate/formic acid (HCOO^−^/HCOOH) or formaldehyde (HCHO). Among these, the production of formic acid or formate is particularly interesting due to their wide versatility in multiples practical applications [43,44,45]. For instance, formate can be used as raw material in chemical, textile, agricultural and pharmaceutical industries. Alternatively, formic acid has demonstrated significant benefits over methanol fuel cell devices [46], including higher open circuit voltage [47] and lower crossover [48]. In addition, the formic acid can be used as hydrogen storage material [49,50].

The electrochemical conversion of CO_2_ to HCOOH/HCOO^−^ is a two-electron process highly favoured on a plethora of non-precious metal surfaces [51] such as Cd [52], In [53,54], Sn [55,56], and Pb [56]. Note that is found that not only these materials in metallic state, but also some of their oxides (e.g., SnOx) are beneficial for CO_2_ reduction since the oxide layer films can inhibit HER [57,58]. Despite Pb and, particularly, Sn have been the most extensively studied systems for formate production, the use of bismuth (Bi) is being considered as a very promising electrocatalyst for obtaining formate (or formic acid) at lower overpotential compared to the above-mentioned metals in aqueous electrolyte. Bi presents interesting properties not only due to its low toxicity and cost, but also due to its higher stability under electrochemical conditions in neutral aqueous media (due to its relatively positive standard reduction potential (Bi^3+^/Bi, 0.308 V) [59]. Also, Bi has been reported to be a poor electrocatalyst for H_2_ evolution because of its positive free energy of hydrogen adsorption [60].

Owing to these interesting properties, the employ of Bi-based electrodes towards ECO_2_R to formate (or formic acid) is increasing significantly [56,61,62,63,64,65,66,67,68,69,70,71,72,73]. For instance, Zhang et al. studied the electrochemical reduction of CO_2_ to formate at room temperature using a high surface area Bi (HAS-Bi) electrocatalyst obtained from the electrochemical reduction of previously prepared BiOCl nanosheets [62]. This electrocatalyst exhibited a good selectivity towards formate with a high faradaic efficiency (around 92%). Also, Zong et al. reported that, using nanostructured Bi dendrite electrocatalyst, electrochemically grown on a carbon paper, displayed a remarkable performance for CO_2_ reduction to formate with a maximum faradaic efficiency of 96.4% and a current density of 15.2 mAcm^−2^ at −1.8 V [63]. Also, the electrode was found to be stable during 10 h of continuous electrolysis. Similarly, Koh et al. demonstrated a high selectivity (∼89% at −0.74 vs RHE) and an efficient conversion of CO_2_ to formate by using a rational design of a hierarchical Bi dendrite catalyst [74]. In addition, a stable performance during ∼12 h operation was achieved with the Bi dendrite catalyst. More recently, new interesting contributions have been reported [64,65,66,67,69,70] including nanosized-Bi electrocatalysts [75,76,77], oxide-derived Bi films [70], Bi nanosheets [78,79], sulphide-derived Bi catalysts [80], and Bi nanoflakes [67], among others, for successfully CO_2_ reduction to formate. However, the preparation of such electrocatalysts requires the use of increasing temperatures, high pressure (autoclave systems are required), non-commercial precursors or in-situ electrochemical reductions which makes their production not easy to be scaled up. Consequently, using a simple, fast and scalable approach is still needed.

In this way, the work aims at the development of an easy and fast procedure for the preparation of carbon-supported Bi nanoparticles under soft experimental conditions. The carbon-supported Bi nanoparticles will be physicochemically and electrochemically characterized and their electrocatalytic properties towards CO_2_ reduction to formate studied in 0.5 M KHCO_3_. Finally, a preliminary analysis of the stability of the Bi-based electrodes was also carried out including microscopic degradation studies.

## 2. Results and Discussion

**Figure 1:**
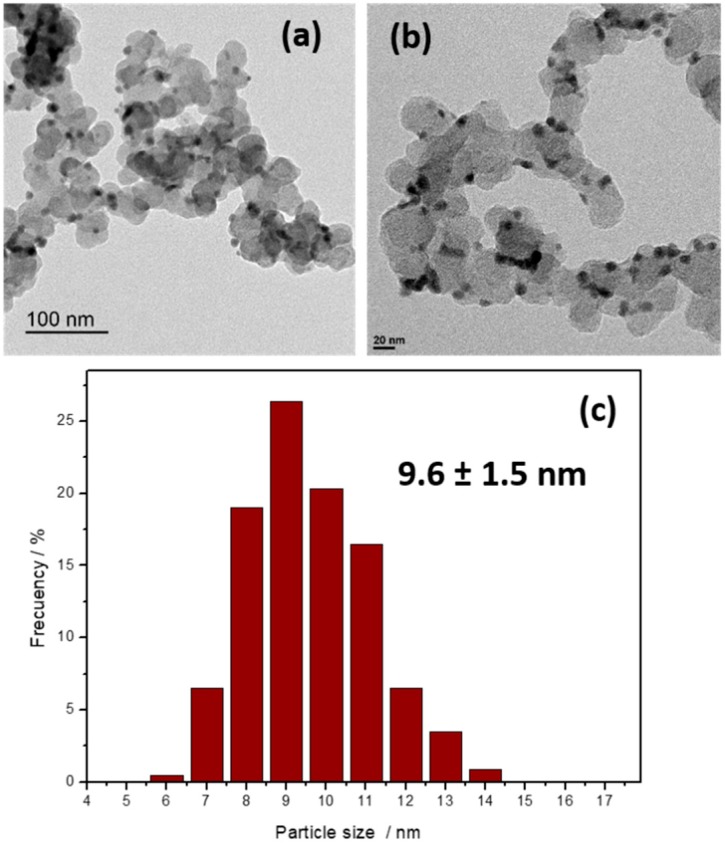
(**a**,**b**) TEM images and (**c**) particle size histogram corresponding to Bi/C prepared with a PVP to Bi ratio of 1.

As mentioned in the Materials and Methods section, syntheses with different PVP to BiCl_3_ stoichiometric ratios (PVP/Bi^3+^: 10, 5, 2, 1, and 0) were tested to be able to form Bi nanoparticles while using the lowest amount of PVP, as the residual presence of PVP may represent a major obstacle for an efficient electroreduction of CO_2_. Figure S1 (see the Supplementary Information) shows some representative TEM images of the samples prepared with different PVP: BiCl_3_ ratios. The results obtained suggest that for PVP to BiCl_3_ ratios of 10, 5, 2, and 1 (see Figure S1a–d), the BiNPs present similar characteristics, that is, a quasi-spherical morphology and a particle size about 10 nm. Interestingly, a low PVP: BiCl_3_ ratio of 1 is enough for controlling the growth of the BiNPs and keeping their particle size in the 10 nm range. This remarkable reduction of the PVP content contribute enormously to the correct and complete washing of the nanoparticles. It is worth recall that clean surfaces are always important for electro-catalysis. On the other hand, as shown in Figure S1e, in the absence of PVP, the sample loses its nanoparticle form and a nanostructured network appears instead of individual nanoparticles with spherical morphology. The formation of this nanostructure may be also interesting, but it is out of our objective of preparing carbon-supported Bi nanoparticles. Once an optimal PVP: BiCl_3_ ratio was determined, carbon-supported BiNPs were prepared. Figure 1 displays some TEM images and the corresponding particle size histogram of Bi/C nanoparticles with a PVP to Bi ratio of 1. The spherical morphology is again clearly observed together with a well dispersion of the nanoparticles on the carbon substrate. The particle size was found to be 9.6 ± 1.5 nm. From AAS measurements, the metal loading was 14.3 wt.%, slightly lower than the nominal one.

XRD analysis of the acetone-washed BiNPs indicates, as expected, the presence of Bi oxides as consequence of the spontaneous oxidation during the washing step/air exposure. Figure S2a,b depict the XRD patterns of the unsupported BiNPs, and Bi/C samples, respectively. Both diffractograms are very similar although, for the Bi/C sample, two small diffraction peaks between 38° and 40°, associated with metallic Bi with a hexagonal crystal structure, are also observed. The quasi-spherical morphology of the BiNPs, as well as an effective surface cleaning, makes them more sensitive to the oxidation. On the other, the XRD pattern of the nanostructured Bi network obtained in the absence of PVP (Figure S2c), clearly shows two sharp peaks at 38° and 40° indicating that, in that case, the sample retains a more metallic state although some features related to Bi oxides are also observed. This finding suggests that the nanostructured meshed network seems more stable towards its oxidation. It is worth noticing that when the nanoparticles were washed with non-anhydrous ethanol instead of acetone, the nanoparticles changed from a black to a white powder, and the diffraction pattern completely modified. The new pattern fits perfectly with that of bismuth carbonate species (bismutite) (Figure S3).

**Figure 2:**
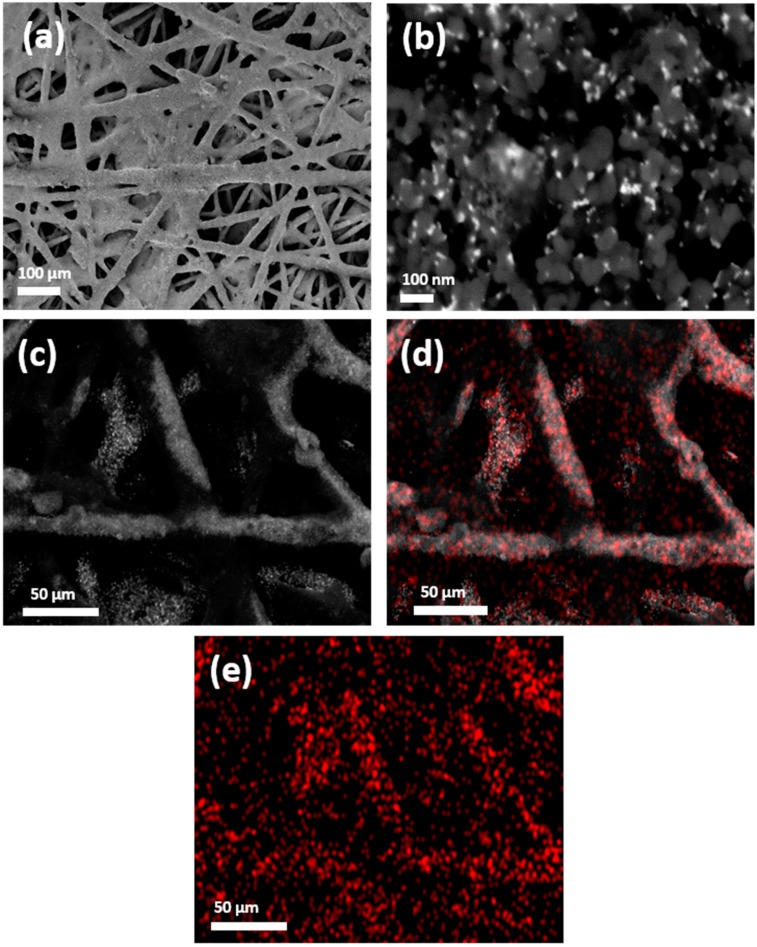
Field emission SEM images of the Bi/C electrode with Bi loading of 0.1 mg cm^−2^: (**a**) magnification ×130, and (**b**) magnification ×100.00 K. Micrographs (**c**–**e**) correspond to SEM-EDX mapping: d and e highlight, in red, the distribution of BiNPs on the carbon substrate.

To get more insight into the oxidation states of the Bi/C sample, XPS measurements were performed. The XPS spectrum of the Bi 4f region (Figure S4) shows the presence of two binding energy contributions at 164.8 (Bi 4f_5/2_) and 159.5 eV (Bi 4f_7/2_) related to Bi^3+^ [81]. In addition, two clear shoulders are observed at 162.6 (Bi 4f_5/2_) and 157.3 (Bi 4f_7/2_) eV denoting the presence of Bi^0^. The Bi^3+^: Bi^0^ ratio was found to be about 94:6, thus confirming that the BiNPs present an oxidised character. Similar features were reported with other Bi-modified electrodes [56,65,67,70]. Also, as previously mentioned in the literature [57], the formation of a Bi oxide stable film could exhibit positive effects on CO_2_ reduction probably by effective inhibition of the hydrogen evolution reaction (HER). As described in the experimental section, the Bi/C sample was used to manufacture Bi-based electrodes. For that, the catalytic ink containing the carbon supported BiNPs was air-brushed onto a carbon paper. Figure 2 shows some representative SEM-EDX images of electrode. The images evidence how the Bi/C ink covers homogenously the carbon Toray fibbers leading to a uniform and compact catalytic layer. At higher magnification, Figure 2b, the good distribution of the BiNPs can be even observed. This homogeneous Bi distribution is further confirmed by the EDX mapping of the plane section of the electrode (Figure 2c–e) from which the uniform distribution of the Bi metal on the carbon substrate with a low metal agglomeration degree is verified.

**Figure 3:**
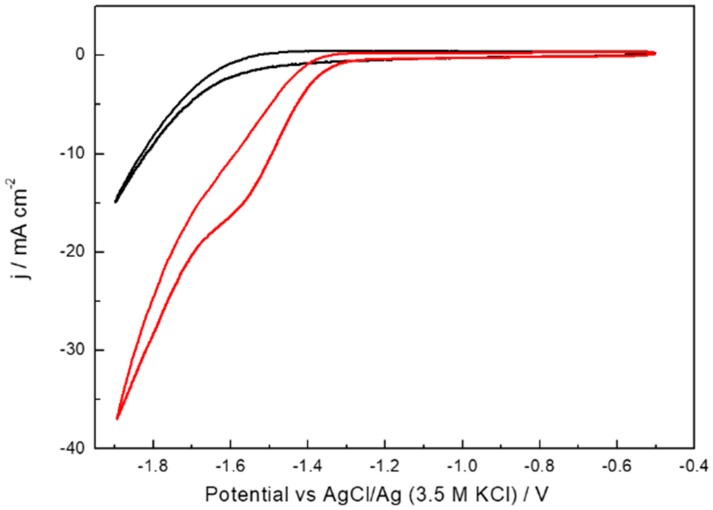
Cyclic voltammetry response of Bi/C electrode with Bi loading of 0.1 mg cm^−2^ in Ar (black line) and CO_2_ (red line) saturated 0.5 M KHCO_3_. Scan rate 50 mV s^−1^.

The electrochemical characterisation of the Bi-based electrodes was performed in an Ar-saturated 0.5 M KHCO_3_ solution, Figure S5 (red line). For sake of comparison, the response of a massive Bi rod is also included, Figure S5 (black line). Both voltammograms display similar voltammetric features which are characterised by a reduction contribution at about −0.6 V vs AgCl/Ag associated with the electrochemical reduction of Bi_2_O_3_ to Bi [80]. In the positive going sweep, the oxidation at about −0.25 V refers to the opposite process, that is, oxidation of Bi to Bi_2_O_3_. For the Bi rod, the HER appears clearly at about −1.4 V vs AgCl/Ag. However, for the Bi/C based electrode, this HER is less evident because of the larger double layer contribution of the carbon substrate. The CO_2_ reduction activity of the Bi/C electrode was evaluated using a CO_2_-saturated 0.5 M KHCO_3_ solution, Figure 3. As expected, the results obtained clearly display the presence of a new and well-defined reduction process starting at −1.3 V and associated with the CO_2_ reduction. A similar feature was observed with the massive Bi rod electrode thus pointing out that, at this potential range, the carbon support does not contribute to the CO_2_ reduction. To better verify the role of the carbon support, Figure S6 displays the response of a Toray paper containing pure Vulcan XC-72R carbon in the absence (Ar saturated 0.5 M KHCO_3_ solution) and in the presence of CO_2_. The results indicate that the activity of the carbon-based electrode towards CO_2_ reduction is very low in comparison with that observed with the carbon supported Bi samples (Figure 3).

**Figure 4:**
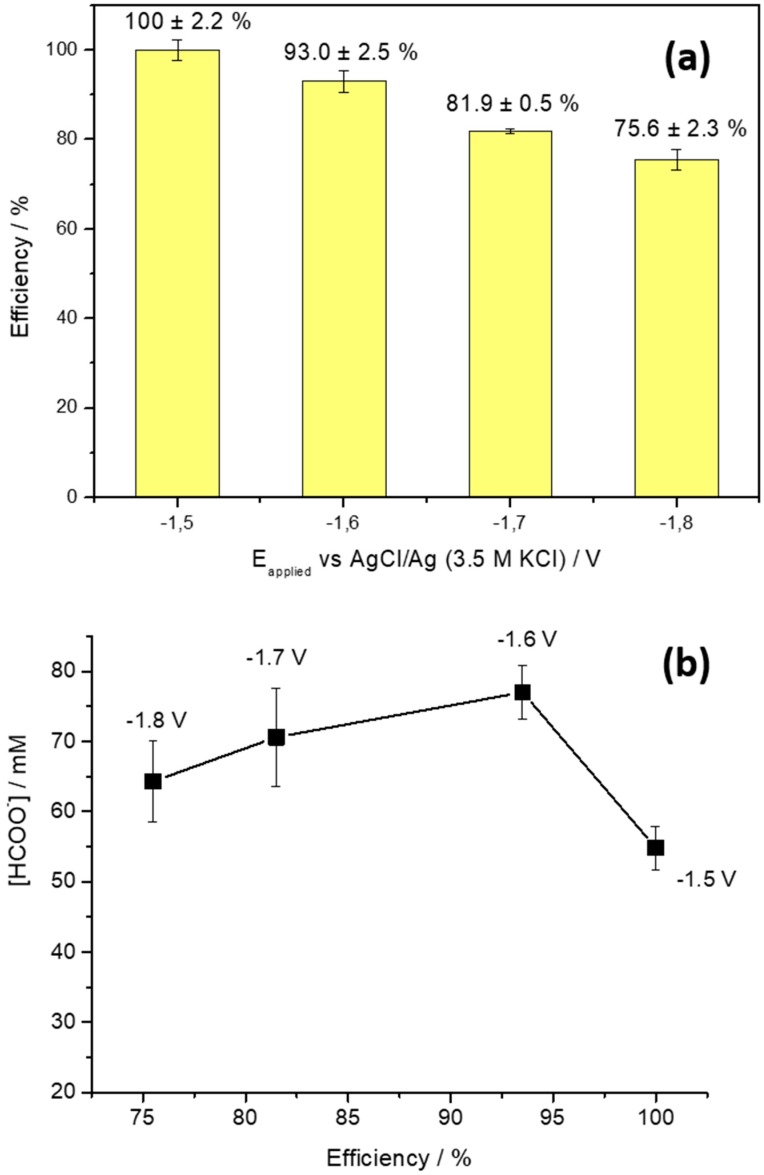
(**a**) Faradaic efficiency for formate production at different controlled potential from −1.5 to −1.8 V. Electrolysis time: 3 h. (**b**) Formate concentration produced after 3 h of electrochemical reduction of CO_2_ at different controlled potentials as a function of Faradaic efficiency.

Subsequently, CO_2_ electroreduction electrolyses were performed in an H-type electrochemical to evaluate the product formation, Faradaic efficiency, and selectivity of the reaction towards formate. For that, consecutive electrolyses were performed at different controlled potentials of −1.5, −1.6, −1.7 and −1.8 V vs. AgCl/Ag in CO_2_-saturated 0.5 M KHCO_3_ solution for 3 h using the experimental setup described below. In these experiments, the current density increases (in absolute value) as the electrolysis potential decreases. Also, the electrolysis current density remains practically constant during the entire experiments (3 h) at all potentials studied (Figure S8). In this work, electrolysis density current values registered ranged between −4 and −12 mA cm^−2^ for −1.5 and −1.8 V, respectively. These values are similar to those reported in previous contributions [65]. Figure 4 summarizes the main findings obtained during the CO_2_ electroreduction electrolysis experiments. Figure 4a shows the Faradaic efficiency as a function of the applied potential. As expected, the faradaic efficiency values decrease for more negative potential values due to the higher contribution of the HER. In any case, a good value of about 76% is found at −1.8 V vs AgCl/Ag.

For lower potential values of −1.5 and −1.6 V for which the contribution of the HER is practically negligible, very high faradic efficiencies were observed towards formate production. Particularly interesting is the ~100% faradic efficiency obtained at −1.5 V. This finding suggests that, at this potential, HER is essentially missing, as previously shown in Figure 3. Obviously, a very minor contribution of the HER is also possible although certainly negligible taking into consideration our experimental error. Numerous electrolyses were carried out at different potentials in order to evaluate the reproducibility of the faradic efficiency values obtained. The standard deviation oscillated between 0.5 and 2.5%, demonstrating the good reproducibility of the measurements. To complete this analysis, it is also important to estimate how the faradaic efficiency evolves during the 3 h experiment. Figure S9 shows the faradaic efficiency values for each hour of reaction. At −1.5 V the FE remains unaltered at the maximum value (100%). A slight decrease in FE is observed between 2 and 3 h of reaction at −1.6 V. This decrease is much more evident at −1.7 and −1.8 V, for which a 20 and 25% loss of FE is observed, respectively. It is worth noting that no obvious detachment of the catalytic layer was observed despite H_2_ bubbles were clearly visible from HER during electrolyses.

Although FE values are relevant for the process, from a practical point of view, analysing the formate concentration produced it is of critical importance. Figure 4b depicts the formate concentration produced after 3 h at different potentials. A maximum formate concentration of 77 mM is found at −1.6 V. This concentration decreases to 70 and 64 mM at −1.7 and −1.8 V, respectively. The lowest formate concentration of 54 mM is found at −1.5 V. The evolution of the formate concentration during the 3-h CO_2_ reduction experiments is shown in Figure S10. In all cases, a linear evolution of the formate concentration is observed. Taking into consideration the FE values and the formate production (in terms of formate concentration), we conclude that, in our experimental conditions, the most convenient potential value is −1.6 V vs. AgCl/Ag.

**Figure 5:**
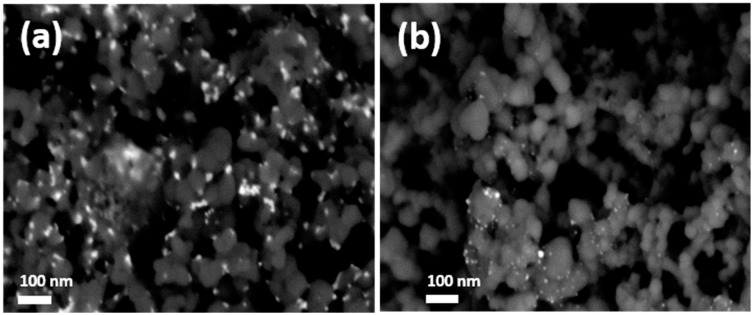
Back-scattered electrons field emission SEM images of the Bi-based electrodes with Bi loading of 0.1 mg cm^−2^: (**a**) as-prepared, and (**b**) after aprox. 70 h in 3-h CO_2_ reduction experiments at different potentials.

Finally, in a preliminary attempt of evaluating the long-term stability of the Bi-based electrodes, we have observed that, after approximately 70 working hours in 3-h CO_2_ reduction experiments at different potential, the electrodes deactivate. To understand the origin of this deactivation, SEM images of the deactivated Bi electrodes were taken, Figure 5 and Figure S11. The SEM images indicate a clear loss of BiNPs in comparison with the freshly prepared electrodes. However, at this point, it is not clear if the detachment of the BiNPs in the electrocatalytic layer is due to the water washing step after each electrolysis and/or to a loss of Bi during the electrochemical/electrolysis experiments. In this respect, it is worth noting that current remains essentially stable during the 3-h CO_2_ reduction experiments (Figure S8) which may suggest that the loss of Bi essentially takes place during the water washing between experiments. 

**Figure 6:**
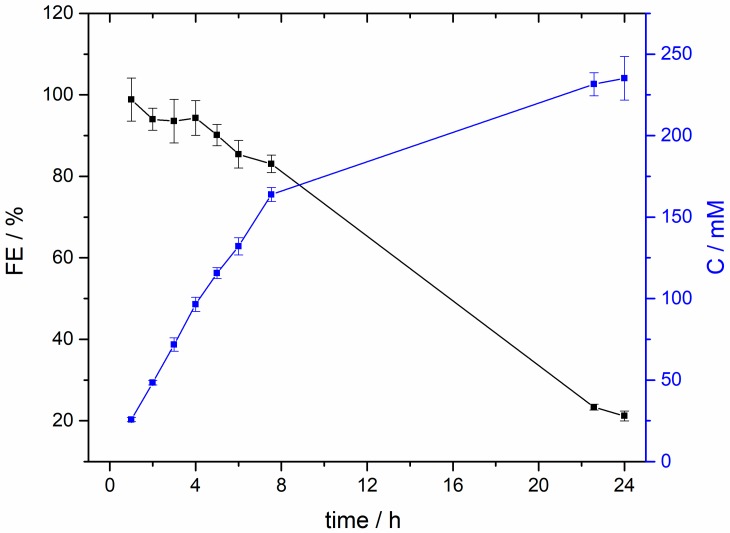
Faradaic efficiency and formate concentration vs time during 24 h CO_2_ electroreduction electrolysis at −1.6 V.

To properly evaluate the stability of the Bi NPs and to find out why the electrode degrades, a long-term (24 h) CO_2_ reduction experiment was carried out at −1.6 V vs. Ag/AgCl in the same H-type electrochemical cell and with a freshly prepared electrode. As shown in Figure S12, the current remains essentially stable during the first 6–7 h. Nevertheless, from this moment, the currents progressively become more negative. At the same time, a more visible generation of bubbles at the surface of the electrode is also observed thus suggesting that HER is becoming predominant in the process. To verify this hypothesis, Figure 6 reports the evolution of the FE and formate concentration during the 24 h experiment. The results confirm that, after 4–5 h, the electrode begins to be less efficient and both FE and formate concentration clearly decay. In particular, the FE decreases from 90–95% (after 5 h) to about only 20% after 24 h. A similar deactivation is progressively observed in term of formate concentration. Thus, whereas in the first 5 h the formate concentration is increasing in about 23 mM/h, this production is significantly reduced to about 6 mM/h during the remaining 19 h. These preliminary results indicate that the Bi electrode strongly deactivates under working conditions. As deduced from the SEM images, Figure 5, this deactivation is attributed to a gradual loss of Bi. This Bi detachment suggests an insufficient interaction between the Bi NPs and the carbon Vulcan substrate. In any case, more work is in progress not only to better understand the mechanism of degradation of the Bi electrodes but also to find new alternatives to improve their durability under working conditions. 

## 3. Materials and Methods

### 3.1. Chemicals and Reagents

BiCl_3_ (99.99%, Aldrich, St. Louis, MO, USA) was used as Bi precursor. N,N-Dimethylformamide (DMF, 99.8%, Sigma Aldrich), polyvinylpyrrolidone (PVP, K30, Mw ~ 55.000, Aldrich), and sodium borohydride (NaBH4 99%, Aldrich) were used as solvent, capping agent and reducing agent, respectively. Vulcan XC-72 carbon powder (CAS No 1333-86-4, sample number GP 3621) was purchased from Cabot Corporation (Boston, MA, USA). The ion exchange cross-linked resin Nafion (5 wt.% in isopropyl/water solution) was purchased from Alfa Aesar (Ward Hill, MA, USA). Cationic ion exchange membrane Nafion 112 was purchased from DuPont, (Wilmington, DE, USA). All other chemicals were purchased from the highest analytical grade available and were used as received without any further purification. All solutions were prepared using MilliQ ultrapure water (18.2 MΩ cm).

### 3.2. Synthesis of Bismuth Electrocatalysts

Bi nanoparticles (BiNPs) were synthesized by chemical reduction of Bi^3+^ in DMF by NaBH_4_ in the presence of PVP as protecting agent. In more details, BiCl_3_ (0.316 g) and PVP K30 (1.116 g) were added into DMF (37.92 g) and sonicated until complete solubilisation. Subsequently, NaBH_4_ (0.116 g) was directly added to the solution under continuous magnetic stirring at room temperature. After the addition of the reducing agent the solution becomes dark, indicating the reduction of Bi^3+^ to Bi^0^. The solution was stirred and alternatively sonicated (Selecta ultrasonic bath operating at 50/60 kHz, 360 W power output, JP Selecta, Barcelona, Spain) for 45 min. Thereafter, the BiNPs were thoroughly washed (four times) and storage with acetone. Different PVP to BiCl_3_ stoichiometric ratios (PVP/Bi^3+^: 10, 5, 2, 1, and 0) were evaluated with the objective of decreasing as much as possible the amount of PVP without affecting the particle size of the Bi nanoparticles. For the preparation of the carbon supported Bi nanoparticles (Bi/C), the chemically reduced BiNPs, in the original DMF suspension, were mixed with appropriated amounts of Vulcan XC-72R carbon powder to obtain a Bi loading of ca. 20 wt.%. After 60 min of continuous magnetic stirring with alternative sonication the Bi/C nanoparticles were precipitated by using acetone and filtered and washed with acetone through a nylon membrane filter of 45 mm (Cat No. MNY045047H, chm by CHMLAB GROUP, Barcelona, Spain). Finally, the sample is dried overnight under vacuum conditions at 45 °C.

### 3.3. Preparation of the Catalytic Ink and Cathode

The catalytic ink was prepared by dispersing the sample in a Nafion solution (perfluorosulfonic acid- PTFE copolymer 5% w/w solution, Alfa Aesar) at a Bi/C:Nafion mass ratio of 80:20. The mixture was then diluted to 2% in absolute ethanol, (EMSURE^®^, Merck, Darmstadt, Germany). Ultrasonic agitation was used to homogenize the ink for at least 30 min. The cathode was prepared by spraying (air-brushing technique) the catalytic ink on a 3 × 3 cm^2^ Toray paper (TGPH-120 from QuinTech, Göppingen, Germany), supported on a hot metallic plate at 90 °C to facilitate solvent evaporation. The Bi loading was 0.1 mg cm^−2^.

### 3.4. Physicochemical Characterisation

Transmission electron microscopy (TEM) images were collected with a JEM-2010 microscope (JEOL, Akishima, Tokyo, Japan) working at 200 kV and with a JEOL JEM-1400 Plus working at 120 kV. The samples were dispersed onto a Formvar-covered copper grid and allowed to evaporate in air at room temperature. For each sample, usually about 200 particles from different parts of the grid were used to estimate the mean diameter and size distribution of the nanoparticles. X-ray diffraction (XRD) patterns were obtained with a D8 Advance diffractometer (Bruker, Billerica, MA, USA) fitted with a copper tube. The optical setup included a Ni 0.5% CuKβ filter in the secondary beam so that only CuKα radiation illuminated the sample (CuKα_1_ = 0.154059 nm and CuKα_2_ = 0.154445 nm). The sample was spread onto a Si wafer and measured in reflection geometry over the 20–90° 2θ range with a step of 0.10° and a counting time of 30 s per step. X-ray photoelectron spectroscopy (XPS) experiments were recorded on a K-Alpha spectrometer (Thermo Scientific, Waltham, MA, USA) using AlKα 1486.6 eV radiation, monochromatised by a twin crystal monochromator and yielding a focused X-ray spot with a diameter of 400 mm, at 3 mA × 12 kV. Deconvolution of the XPS spectra was carried out using a Shirley background. Scanning electron microscopy (SEM, S-3000 N microscope, Hitachi, Krefeld, Germany, working at 20 kV with a Bruker Xflash 3001 X-ray detector for microanalysis) was employed to analyse the morphology of the electrocatalytic layer of the manufactured electrodes. High resolution SEM images were obtained using a field emission scanning electron microscopy (FESEM) (model Merlin VP Compact, ZEISS, Oberkochen, Germany). The metal (Bi) loading was experimentally analyses by atomic absorption spectroscopy (AAS) using a SpectrAA-220 FS instrument (Varian, Palo Alto, CA, USA). For that, a known amount of the carbon supported Bi nanoparticles was firstly treated in nitric acid and then filtered. Finally, the sample was conveniently diluted using a 2 wt% HNO_3_ water solution.

### 3.5. Electrochemical Characterisation

The electrochemical characterisation of the Bi-based electrodes was performed in a three-electrode configuration glass cell in Ar or CO_2_-saturated 0.5 M KHCO_3_ (99.7%, Sigma Aldrich) solution using a platinum wire and an AgCl/Ag (3.5 M KCl) as counter and reference electrodes, respectively. Cyclic voltammetry (CV) experiments were performed using a PGSTAT302N system (Metrohm Autolab B. V., Utrecht, Netherlands). All CV measurements were performed at 25 ± 1 °C. Currents were normalised by the geometric area of the electrodes. For sake of comparison, a massive Bi rod (bismuth rod, 11 mm diameter, 99.99% (metals basis), Alfa Aesar) was also employed. CO_2_ electroreduction electrolyses were performed in an H-type electrochemical cell with divided compartments through a cationic ion exchange membrane (Nafion 112). A CO_2_-saturated 0.5 M KHCO_3_ solution (CO_2_ continuous flux of 200 mL min^−1^) was used as catholyte. The anolyte was a 1.0 M KOH (85%, Panreac, Barcelona, Spain) solution. The Nafion 112 membrane was previously activated in 0.1 M NaOH for 24 h. A long platinum wire immersed in the anolyte acted as counter electrode. An AgCl/Ag (3.5 M KCl) electrode placed in the catholyte was reference electrode. The CO_2_ electroreduction electrolyses were carried out by chronoamperometry measurements at controlled potential between −1.5 V and −1.8 V for 3 h using the PGSTAT302N. The conversion of CO_2_ to formate was followed by ionic chromatography (690 Ion Chromatograph Ω Metrohm, Herisau, Switzerland), with conductivity range of 1000 μScm^−1^ and sensitivity of 200 μScm^−1^), with an AS9-HC (Dionex, Sunnyvale, CA, USA) coupled to an UV-Vis detector. The mobile phase consisted of 4.5 mM Na_2_CO_3_ with pH < 12. A calibrate curve was initially carried out from 0 to 60 ppm in 0.005 M KHCO_3_, that is, a 100 times dilution of the catholyte. The calibration curve is included in the Supporting Information (Figure S7).

## 4. Conclusions

An easy, fast and scalable methodology for the preparation of BiNPs at room conditions has been described. The nanoparticles have been characterised, presenting a particle size of about 10 nm, an oxidised state, and a good dispersion on the carbon substrate. Their electrochemical characterisation shows similar features than that observed with a massive Bi rod. The electrocatalytic properties of the nanoparticles towards CO_2_ reduction to formate have been evaluated. As expected, the sample displays a high activity and selectivity towards formate. In 3-h CO_2_ electroreduction electrolyses, we have found that the optimal electrode potential is −1.6 V vs AgCl/Ag at which the concentration of formate was about 77 mM with a Faradaic efficiency of 93 ± 2.5%. A ~ 100% Faradaic efficiency was observed at a lower potential (−1.5 V vs AgCl/Ag) at expenses of lowering the formate concentration (~55 mM). The stability of the Bi-based electrodes has been preliminary tested. Unfortunately, our findings show that, after about 70 h (in 3-h electrolysis experiments at different potentials), the electrode deactivates. This deactivation is attributed to a gradual loss of Bi as shown by SEM/EDX analyses. A 24 h experiment suggests that the degradation takes place under working conditions. More work is in progress to understand this degradation mechanism and to enhance the durability of the Bi electrodes. Future research also involves the use of these carbon supported Bi nanoparticles to prepare Gas Diffusion Electrodes (GDEs) as previously described with Sn nanoparticles [82].

## Supplementary Materials

The following are available online at https://www.mdpi.com/1420-3049/24/11/2032/s1, Figure S1: TEM images of unsupported BiNPs synthesized from different stoichiometric PVP to BiCl_3_ ratios, Figure S2: XRD diffractograms of BiNPs prepared with a PVP to Bi ratio 1, Figure S3: XRD diffractogram of BiNPs prepared with a PVP to Bi ratio of 1 and washed with non-anhydrous ethanol instead of acetone, Figure S4: High resolution XPS spectra recorded of Bi 4f region of the Bi/C electrodes, Figure S5: Cyclic voltammograms obtained in Ar-saturated 0.5 M KHCO_3_ solution at a scan rate of 50 mV s^−1^ saturated with a (red) Bi/C electrode (Bi loading: 0.1 mg cm^−2^) and with a (black) massive Bi rod, Figure S6: Cyclic voltammograms in Ar (black) and CO_2_ (red) saturated 0.5 M KHCO_3_ solution at a scan rate of 50 mV s^−1^ with a Vulcan XC-72R carbon electrode (without Bi), Figure S7: Formate calibrate curve obtained from ionic chromatography analysis, Figure S8: Chronoamperometric measurements at relevant potentials for 3 h, Figure S9: Faradaic efficiency for formate production at different controlled potential as a function of time, Figure S10: Formate concentration vs time at different potentials, Figure S11: Back-scattered electrons field emission SEM images of the Bi-based electrodes as-prepared, and after aprox. 70, Figure S12: Chronoamperometric measurement during 24 h.
