# Supplementary material for: Electrochemical Reduction of CO2 to Formate on Easily Prepared Carbon-Supported Bi Nanoparticles
Source: Molecules. 2019 May 28;24(11):2032. doi: 10.3390/molecules24112032 (PMC6600365; doi:10.3390/molecules24112032)
Supplement: Supplementary file 1 [file molecules-24-02032-s001.pdf]

Supporting Information

Electrochemical Reduction of CO_2 to Formate on Easily Prepared Carbon-Supported Bi Nanoparticles

Beatriz Ávila-Bolívar, Leticia García-Cruz, Vicente Montiel and José Solla-Gullón *

Instituto de Electroquímica, Universidad de Alicante, Apartado 99, 03080 Alicante, Spain; beatriz.bolivar@ua.es (B.A.-B.); leticia.garcia@ua.es (L.G.-C.); vicente.montiel@ua.es (V.M.)

* Correspondence: jose.solla@ua.es

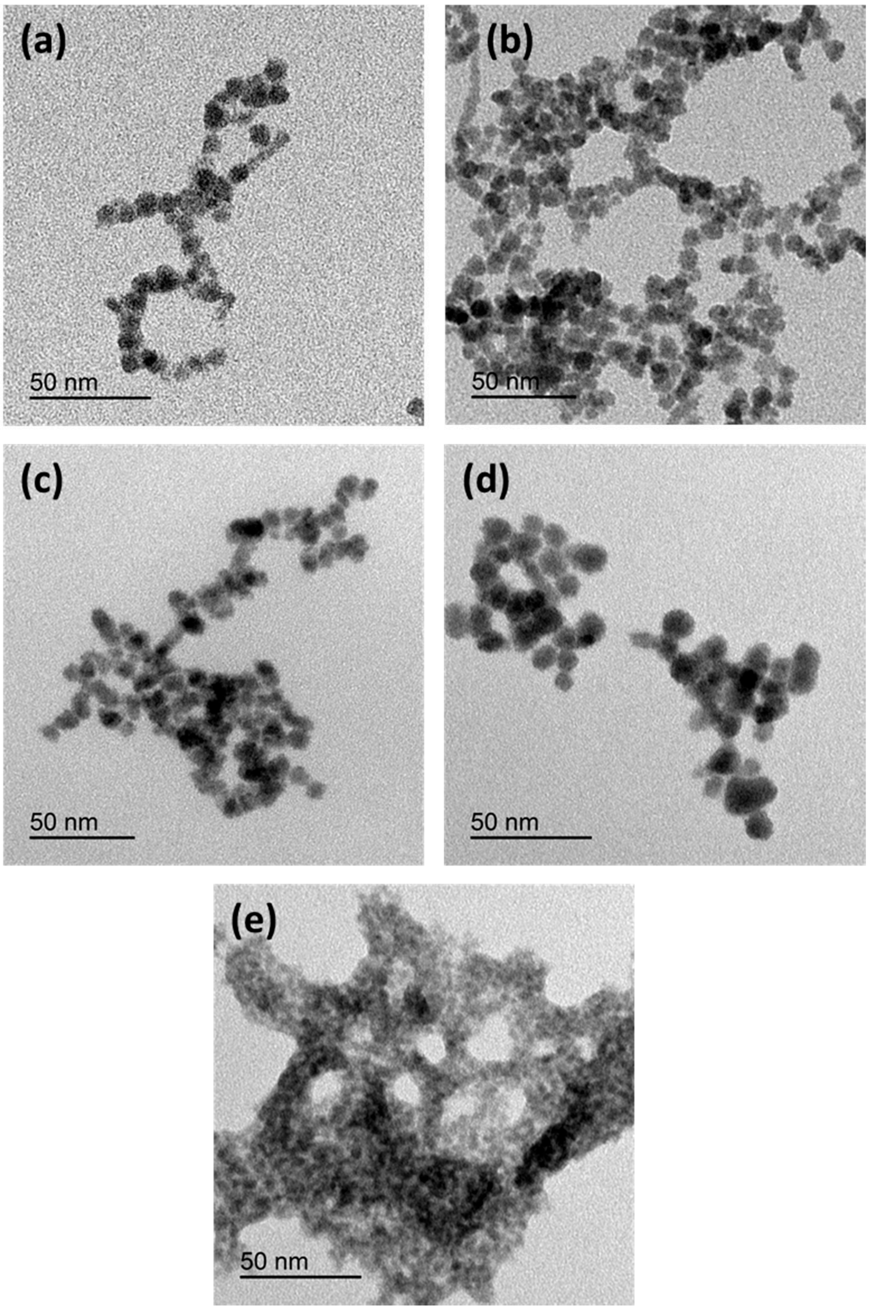

Figure S1. TEM images of unsupported BiNPs synthesized from different stoichiometric PVP to $BiCl_3$ ratios of (a) 10, (b) 5, (c) 2, (d) 1, and (e) 0.

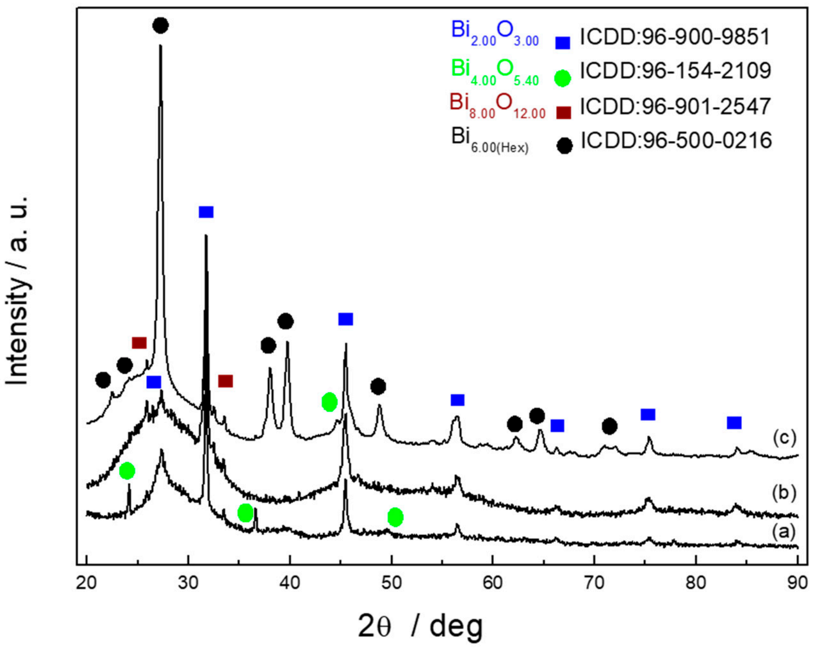

Figure S2. XRD diffractograms of BiNPs prepared with a PVP to Bi ratio of 1: (a) unsupported BiNPs and (b)Bi/C. (c) XRD diffractogram of unsupported BiNPs prepared with a PVP:Bi ratio of 0:1.

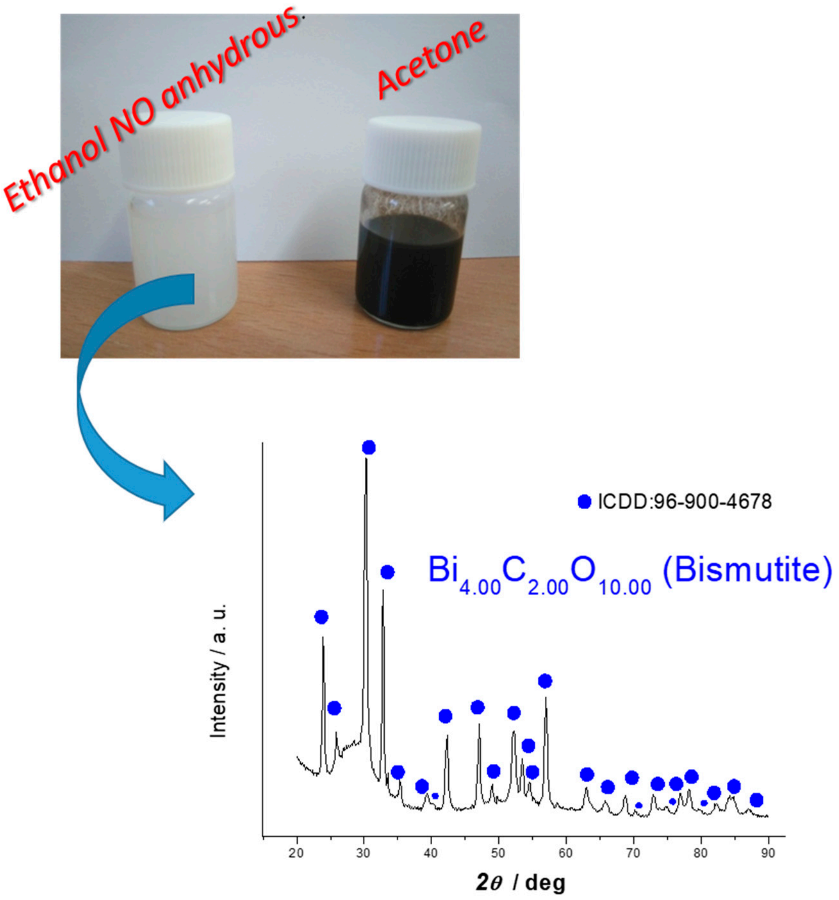

Figure S3. XRD diffractogram of BiNPs prepared with a PVP to Bi ratio of 1 and washed with non-anhydrous ethanol instead of acetone.

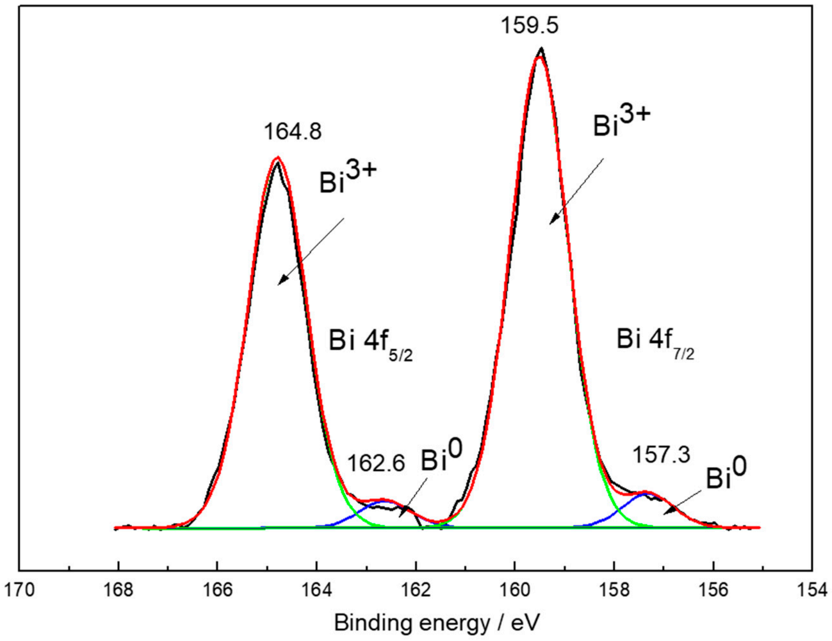

Figure S4. High resolution XPS spectra recorded of Bi 4f region of the Bi/C electrodes.

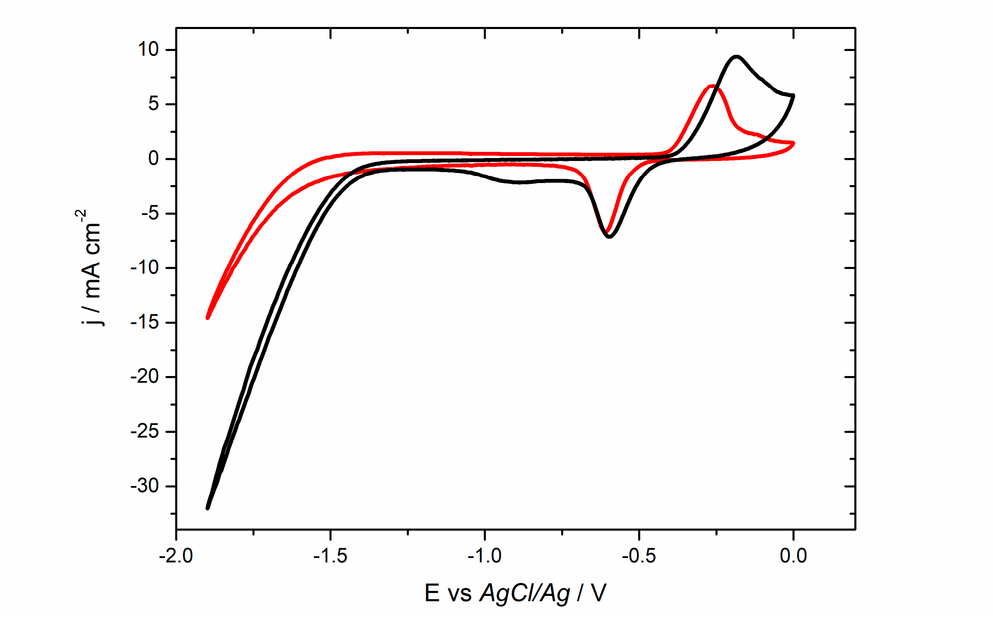

Figure S5. Cyclic voltammograms obtained in Ar-saturated 0.5 M $KHCO_3$ solution at a scan rate of 50 mV s^{-1} saturated with a (red) Bi/C electrode (Bi loading: 0.1 mg cm^{-2}) and with a (black) massive Bi rod.

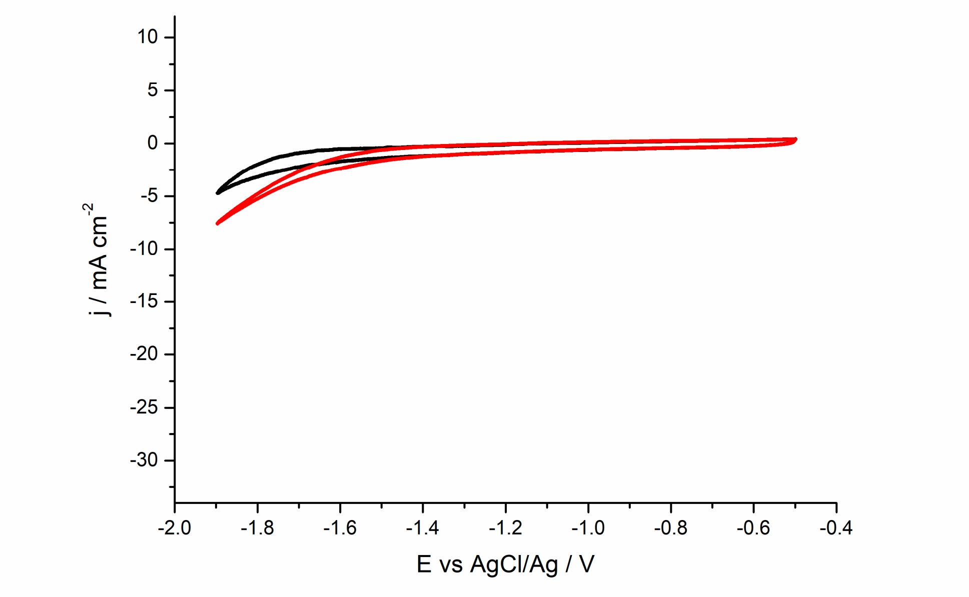

Figure S6. Cyclic voltammograms in Ar (black) and CO_2 (red) saturated 0.5 M $KHCO_3$ solution at a scan rate of 50 mV s^{-1} with a Vulcan XC-72R carbon electrode (without Bi).

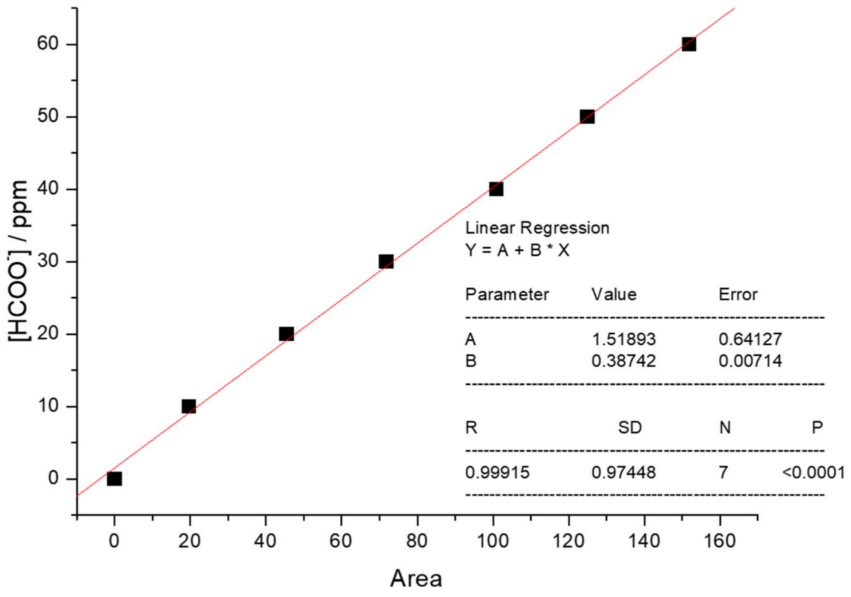

Figure S7. Formate calibration curve obtained from ion chromatography analysis.

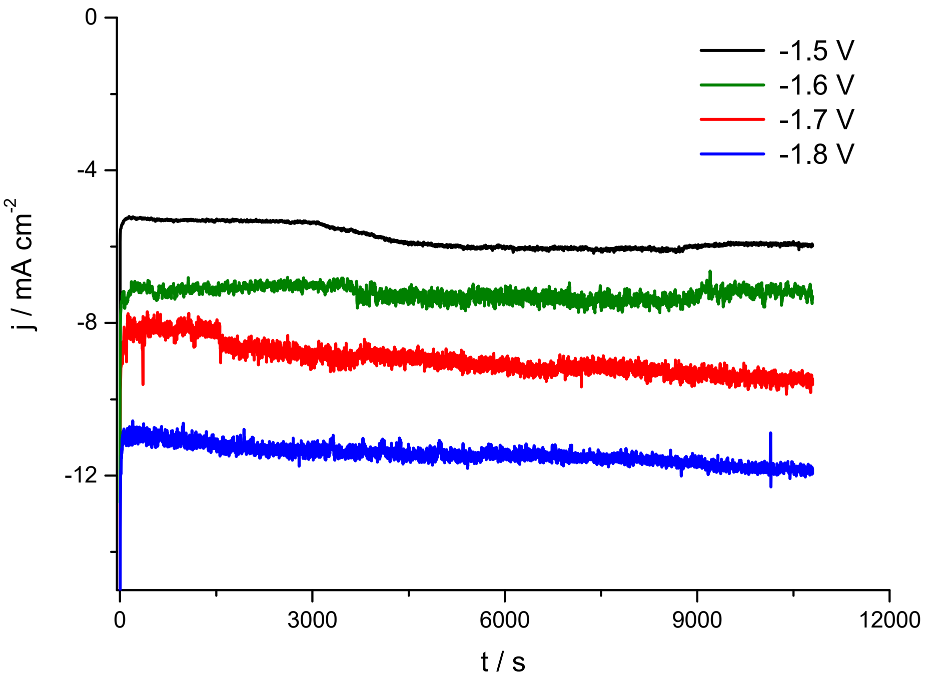

Figure S8. Chronoamperometric measurements at relevant potentials for 3 h.

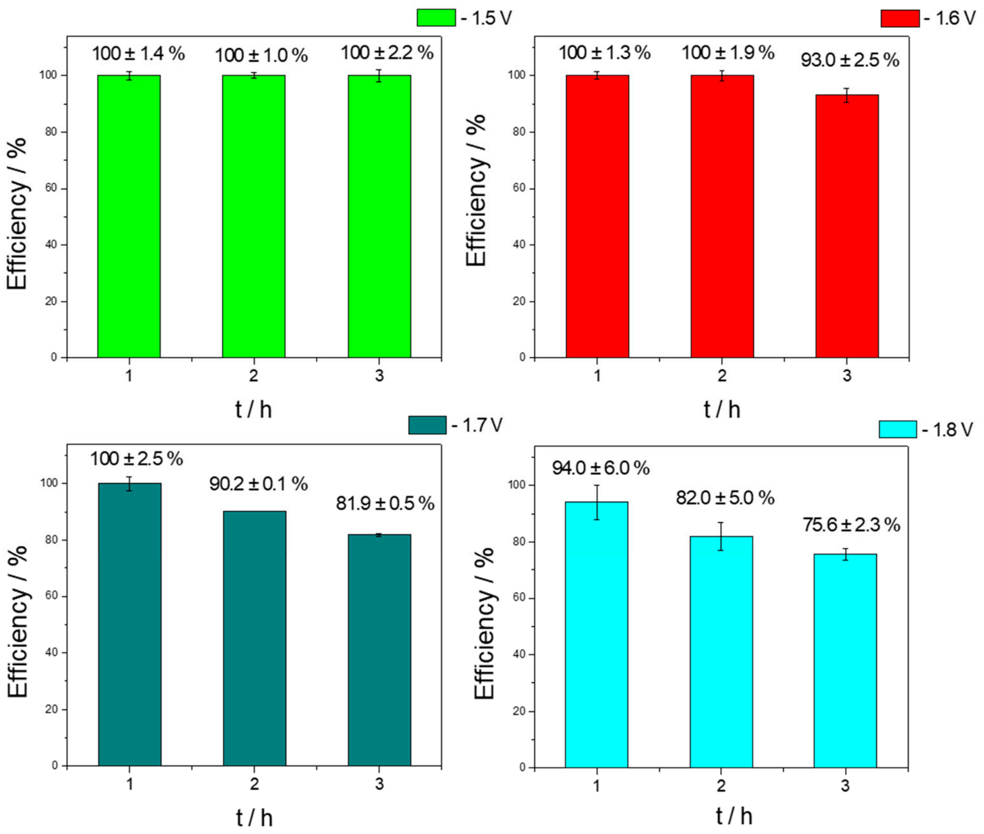

Figure S9. Faradaic efficiency for formate production at different controlled potential as a function of time.

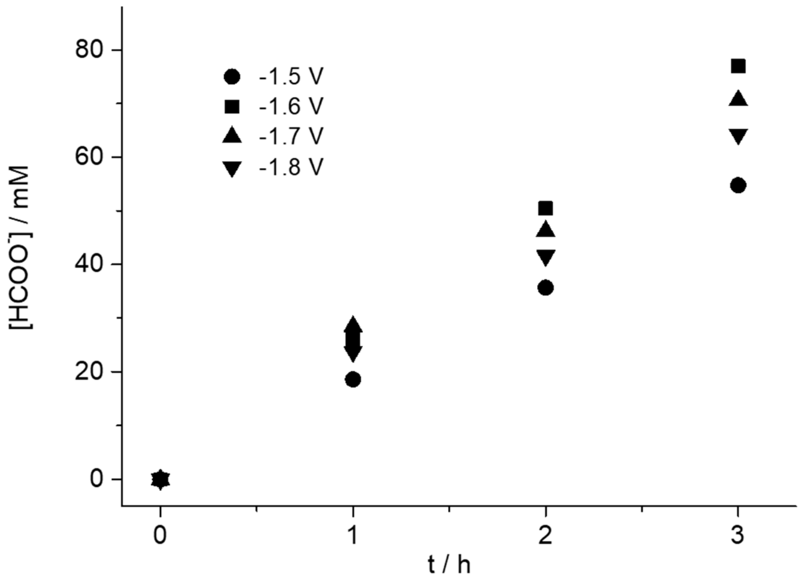

Figure S10. Formate concentration vs time at different potentials.

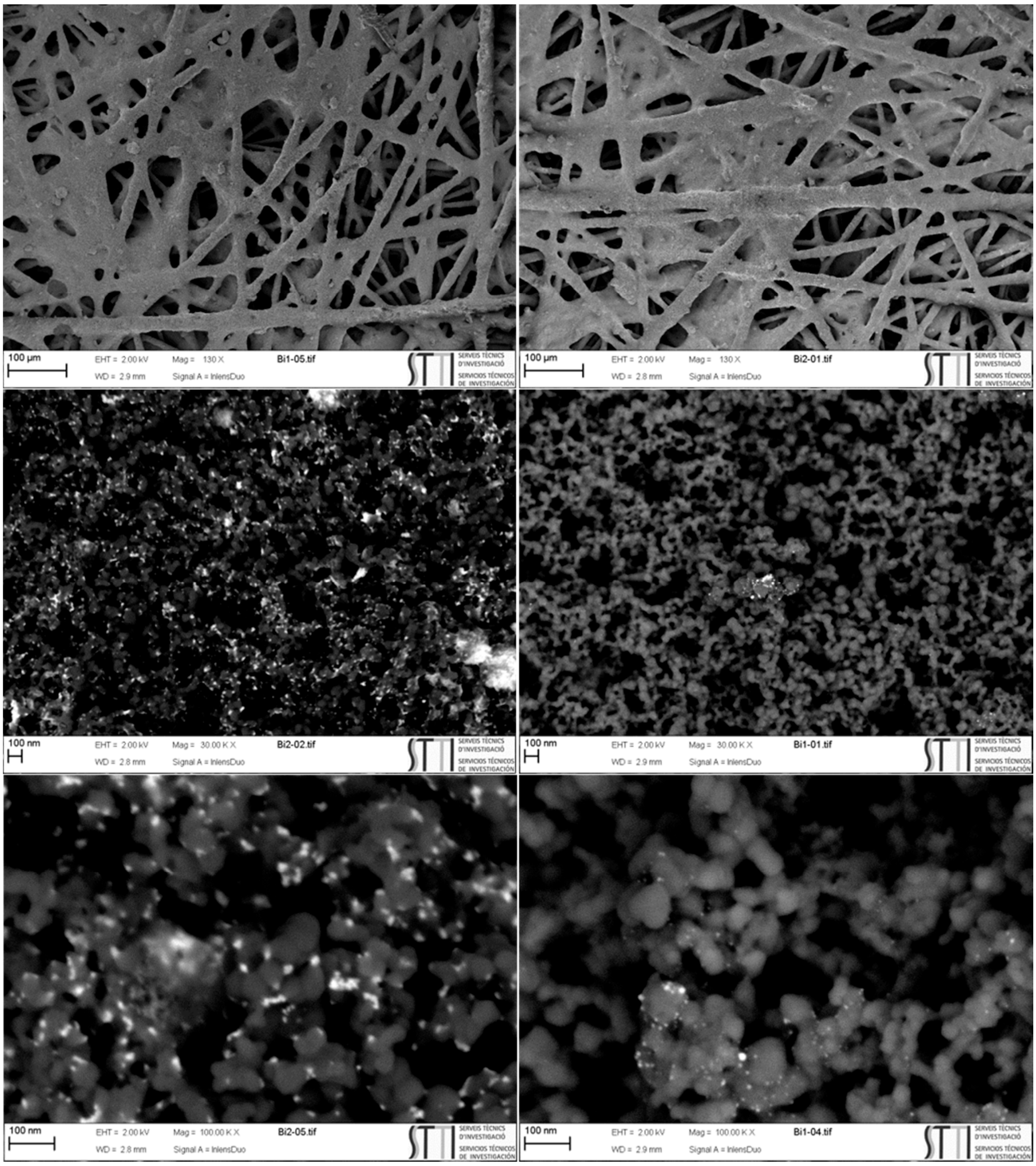

Figure S11. Back-scattered electrons field emission SEM images of the Bi-based electrodes with a Bi loading of 0.1 mg cm^{-2}, (left column) as-prepared, and (rigth column) after aprox. 70 hours in 3-hour CO_2 electrolyses at different controlled potentials.

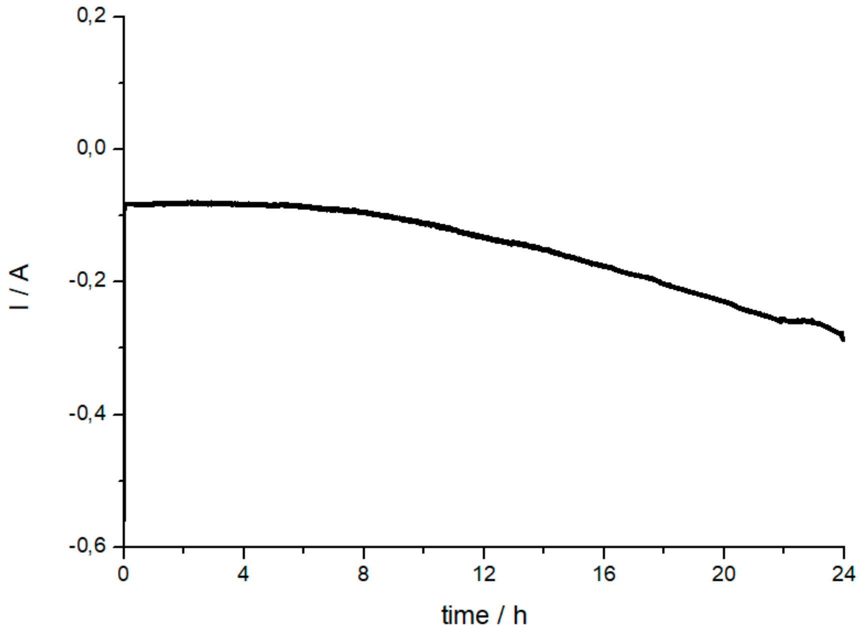

Figure S12. Chronoamperometric measurement at -1.6 V vs. *AgCl/Ag* during 24 h.
